# Legal judgment prediction via dynamic task graph and keyword-aware contrastive learning

**DOI:** 10.1371/journal.pone.0357463

**Published:** 2026-09-10

**Authors:** Jiawei Li, Shengjia Zhang

**Affiliations:** 1 Department of Network Engineering, Henan University, School of Software, Kaifeng, China; 2 Department of Computer Science and Technology, Henan University, International School of Technology, Kaifeng, China; Hubei University, CHINA

## Abstract

Legal judgment prediction (LJP) aims to predict judgment results based on the factual descriptions of criminal cases and is gradually becoming a popular research topic in the legal field. Case factual descriptions contain a large amount of keyword information, and there are complex dependencies among the subtasks. For example, law article prediction can guide charge prediction and term of penalty prediction. However, most of the previous methods often failed to take advantage of the effective keywords that are commonly present in case descriptions, or failed to consider the interrelationships between sub-tasks. To address the shortcomings, we propose a multi-task legal judgment prediction framework based on Dynamic Task Graph and Keyword-aware Contrastive Learning, termed DTG-KCL. Specifically, we design a sample-level method of dynamic task relationship graph convolution to encode task relationships from the perspective of each input case description. Subsequently, we enhance the ability to distinguish confusing charges by developing a more profound and novel keyword fusion mechanism based on soft keyword representation and an approach of keyword-aware contrastive learning. Our model effectively improved the performance of the sub-tasks. Experimental results on two real legal datasets demonstrated that our model had a significant advantage over the five methods in terms of prediction performance. Code can be obtained from: https://github.com/YiLisiForever/DTG-KCL.

## Introduction

**Fig 1:**
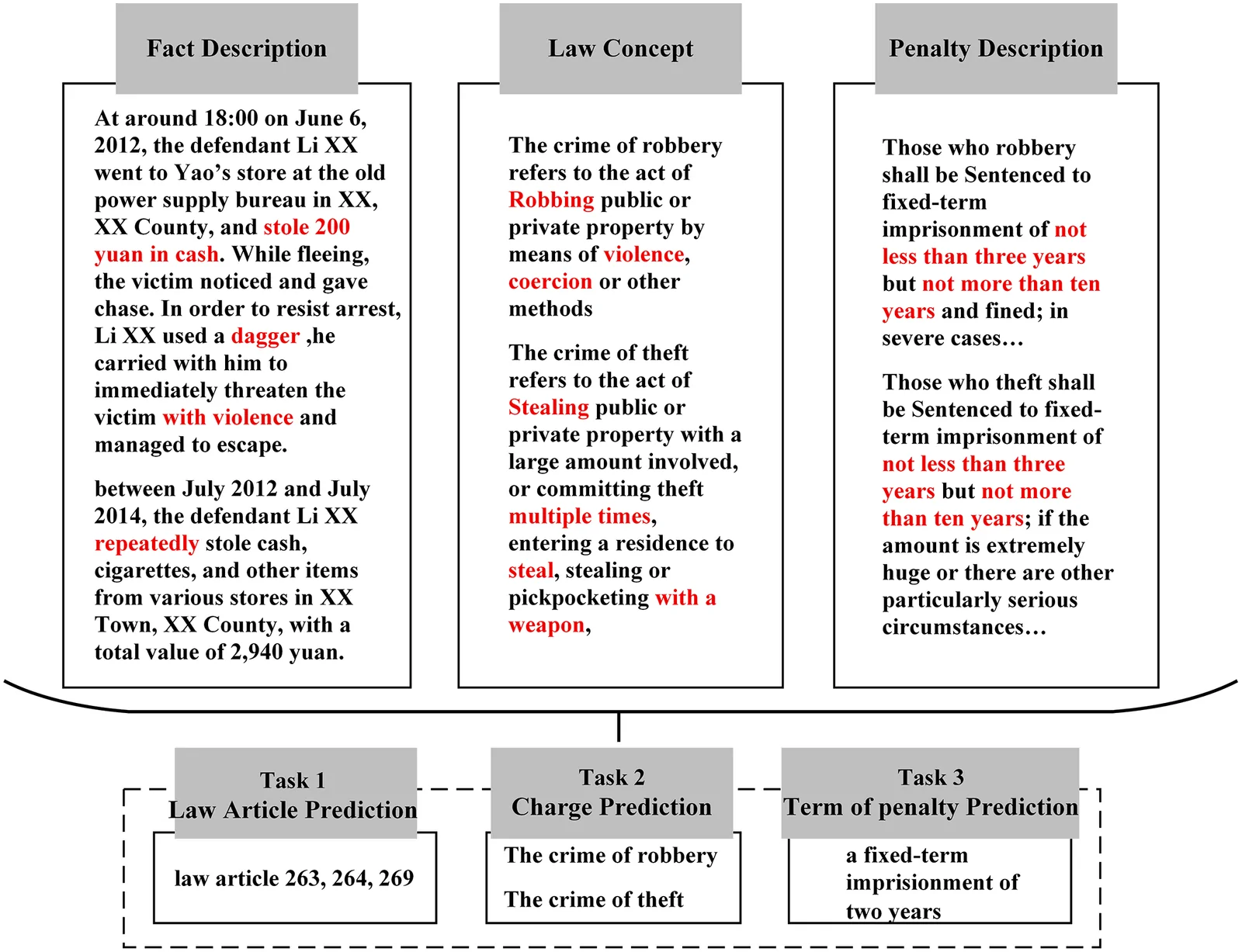
Diagram of legal judgment prediction (LJP).

In general, a classic legal judgment prediction (LJP) consists of multiple subtasks, namely law article prediction, charge prediction, and term of penalty prediction. As shown in (Fig 1), a typical LJP model extracts the features of case from a factual description, a law concept, and a penalty description, and then identifies the corresponding law articles, charges, and penalties based on these respective features. Therefore, the LJP model can be fundamentally modeled as a multi-task problem, and its results can serve as a reference, which is helpful in effectively reducing the workload of lawyers and judges.

LJP also has great potential for use in a legal assistant system. For example, LJP enables non-legal professionals to obtain preliminary judgment results, while legal professionals can regard it as a useful reference. Furthermore, by recommending relevant legal articles, it enhances interpretability and transparency for both legal and non-legal professionals.

In fact, the complexity of judicial decisions makes it extremely challenging to design an effective legal judgment prediction (LJP) system. The methods commonly used in earlier studies heavily relied on domain expertise and time-consuming manual annotations, and had significant limitations in scalability and efficiency. Therefore, deep neural network models gradually replaced them and became the dominant paradigm in LJP research. For example, although TextCNN proposed by Kim [1] applied convolutional neural networks to natural language processing tasks, its ability to extract case information is insufficient for rigorously structured legal documents. Similarly, although the deep gated network proposed by Chen et al. [2] improved the term of penalty prediction, it did not address the issue of law article prediction and charge prediction in LJP.

Overall, the existing legal judgment prediction studies have three major challenges.

First, there is the limitation of pre-fixing subtask relationships. Zhong et al. [3] introduced a pre-defined directed graph to model the relationships among sub-tasks. Yang et al. [4] propose the multi-perspective bidirectional feedback network (MPBFN). Nevertheless, these works rely on prior knowledge, and when there are more subtasks and more dependencies, pre-designing all topological dependencies is inefficient.

Second, some charges are easily confused in practice. For example, for “smuggling, trafficking, transporting, and manufacturing drugs” and “illegally possessing drugs,” the factual descriptions of these two charges may differ by only a few keywords. The key distinction between the two lies in whether the drugs are in “static possession” or “mobile diffusion,” yet the judgment results for the two charges are completely different, especially when aggravating circumstances exist. Distinguishing such ambiguous information can significantly enhance both the accuracy and interpretability of charge prediction.

Third, there is inaccurate modeling of case keywords and insufficient fusion between keyword representations and case representations. LADAN proposed by Xu et al. [5] applies TF-IDF [6] to extract law article information for constructing a graph convolutional neural network, and MVE-FLK proposed by Yang et al. [7] also uses TF-IDF to extract keywords. However, TF-IDF is inaccurate for modeling law articles in some cases and can easily omit high-frequency key information. As shown in the Table 1, the keywords extracted by MVE-FLK do not adequately capture the critical information of the corresponding cases. Furthermore, MVE-FLK merely concatenates or adds the case representation and keyword representation for fusion, without the case representation guiding the keyword representation. Moreover, this approach does not significantly enhance the model’s ability to discriminate between confusing charges.

**Table 1:** Keywords accuracy comparison.

Charge	MVE-FLK Keywords	Our Keywords
Abusing of power	Losses, official, powerlessness, authority, cause, surpass, staff, benefits, people, country	Authority, abuse, overstepping, exceeding, Randomly, favoritism, fraud

To solve the above challenges, we propose DTG-KCL (Dynamic Task Graph and Keyword-aware Contrastive Learning) for simultaneously processing multiple sub-tasks in LJP. In summary, the three main contributions of this paper are as follows:

- Under the guidance of legal experts, we designed a keyword library for charges. Compared with using NLP methods to automatically extract keywords, this approach is more accurate and effective.
- To automatically extract subtask relationships, we design a sample-level method of dynamic relationship graph convolution. We achieve this by allowing the weights of the adjacency matrix in the graph convolution to change dynamically based on the input cases, rather than having all cases share the same set of weights, thereby forming a dynamic graph. As a result, different cases activate different task dependency paths. This method can effectively select important and informative dynamic relationships among sub-tasks.
- To address the problem of distinguishing confusing charges, we propose a keyword-aware contrastive learning approach, which includes soft keyword representation fusion with convolutional representation and keyword-based supervised contrastive learning. Unlike previous methods that simply combine several classic encoders while ignoring keyword encoders; to obtain more effective case information from charge keywords, we use a case-representation-guided auxiliary task to fuse soft keyword representations with case graph convolution representations. We then perform supervised contrastive learning using similarity of charge keyword, treating cases with similar keywords but different charges as difficult negative samples. By employing WeightSupConLoss, we enlarge the distance between easily confused cases in the feature space, thereby enhancing the model’s ability to distinguish confusing charges.

## Related works

1. Legal judgment prediction

The early work on legal judgment prediction (LJP) primarily employed mathematical and statistical algorithms [8–10]. However, these approaches often depended heavily on domain expertise and labor-intensive manual annotation, rendering them time-consuming and difficult to scale. In recent years, the advancements in deep learning have led to an increasing number of researchers exploring the use of deep neural networks to solve the LJP problem. Some studies have focused on how to better leverage the semantic information of law articles and charges. For example, Liu et al. [11] proposed a method that leverages the definitions of legal articles and charges to transform the representation of case facts into multiple label-specific representations for predicting legal articles and charges. Since similar charges have close descriptions in law articles and previous work performed poorly on few-shot charges, Hu et al. [12] introduced manual features of charges to improve performance on few-shot charges.

Other related studies have focused on extracting legal attributes or adding additional input information to enhance the performance of LJP, such as fact descriptions, defendant information, court opinions, etc. For example, Xu et al. [13] integrate the keywords related to the charges into the multi-task learning model. Furthermore, Li et al. [14] improved the accuracy of sentence prediction by extracting case features. Yang et al. [7] proposed MVE-FLK, which enhances model performance by encoding the keywords and combining them with multiple advanced models. Guo et al. [15] extract fine-grained entity-relation triples from each fact description, then leverage them to construct a Fine-Grained Element Graphs (FEGs) to represent fact descriptions and applies Graph Attention Network (GAT) to learn graph representation for subsequent prediction tasks.

2. Multi-task learning in LJP

The purpose of multi-task learning is to jointly learn multiple related tasks and integrate the knowledge from tasks, thereby improving the generalization performance of all related tasks [16,17].

it has been widely applied in numerous applications across different fields of natural language processing [3,4,18].

In the field of LJP, considering the natural dependencies among sub-tasks (law article prediction, charge prediction, term of penalty prediction), researchers have begun to explore multi-task learning frameworks. Zhong et al. [3] models the relationships between sub-tasks as a directed acyclic graph (DAG) that improves prediction performance by explicitly modeling the dependencies between sub-tasks. Building on this work, Yang et al. [4] further designed a bidirectional long short-term memory (Bi-LSTM) network with an attention mechanism and combined this framework with the dependencies among sub-tasks, proposing the multi-perspective bidirectional feedback network (MPBFN). This network designs a multi-perspective forward prediction and backward verification mechanism, integrating the prediction results of sub-tasks into the network, thereby enabling bidirectional information exchange between sub-tasks.

However, such approaches rely on priori knowledge. When there are more sub-tasks or more dependencies, pre-designing all topological dependencies becomes inefficient. Therefore, the DAP (Dependencies Auto-learning Predictor) proposed by Yao et al. [18] uses an attention mechanism to automatically extract sub-task relationships based on task similarity. Qin et al. [19] proposed a method that explicitly combines legal judgment prediction with legal document retrieval. It integrates legal judgment prediction with legal document retrieval.

In DAP, the task relationship modeling based on attention uses the bilinear matching to calculate the global similarity between all task pairs as the correlation between tasks, thereby learning the task relationships. This paper employs sample-level adaptive graph neural networks for multi-task modeling. Each task is regarded as a graph node, and edge weights are dynamically generated through a two-layer transformation, followed by neighbor representation aggregation. This approach not only has stronger nonlinear fitting capabilities but also allows different dependencies of the same task to vary across different samples. The model can adaptively adjust the degree of interaction based on the input content, enabling it to capture more fine-grained interaction patterns, rather than merely calculating the global shared similarity.

3. Contrastive learning for confusing law article

Contrastive learning aims to learn effective data representations by pulling semantically close neighbors together and pushing apart other non-neighbors. Because it can efficiently utilize training data without requiring annotations, contrastive learning has been widely applied in computer vision [20–23] and natural language processing [24,25].

Commonly, in visual representation, neighbors are formed by performing transformations on the same image [20]. Similarly, in text representation, data augmentation techniques can be employed to generate text neighbors from a given text sequence [25,26]. There are also supervised contrastive learning models [22,24].

LADAN [5] proposed by Xu et al. extracts discriminative and representative information from all law articles, which is then used to distinguish easily confused cases. However, the ability to distinguish easily confused cases relies on the internal information flow of the model, making it less explainable compared to supervised contrastive learning.

CL4LJP [27] proposed by Zhang et al. treats the law article corresponds to the fact description of a certain case as a positive pair, and other law articles from the same chapter are used to construct negative pairs. However, the similarity between law articles is also crucial information. Generally, cases with similar keywords are more easily confused, and contrastive learning constructed in this way cannot leverage the similarity of keywords to distinguish easily confused cases. This paper improves the supervised contrastive learning method used by CL4LJP. The contrastive learning method used by CL4LJP can only handle two categories. Therefore, this method is improved by weighting the difficult negative samples, which enables weighted processing of three categories and enhances the discriminative ability of the model.

4. Large language models in LJP

The emergence of large language models (LLMs) makes a significant breakthrough in Legal judgment prediction. Faria. [28] et al conducted an artificial verification and proved that GPT-4 can accurately complete the extraction of information from legal documents, demonstrating the revolutionary potential of LLM in legal information processing. Collaborative Model Reasoning is a method that enhances the quality of reasoning through the collaborative work of multiple models [29–31]. Jiang et al. [32] proposed AgentsBench, which uses multiple large language model-driven intelligent agents to simulate the deliberation process of the Chinese jury system, better reflecting authenticity and fairness.

### Methodology

1. Problem formulation

In this section, some basic definitions will be introduced.

**Definition 1** (Fact Description):

We use Thulac [33] to tokenize the factual descriptions of cases. Thus, each factual description can generate a sequence of words $S\;=\;\{s_{\text{1}},s_{\text{2}},...,s_{n}\}$, where n is the number of words in the vocabulary sequence corresponding to the factual description, and $s_{i}$ denotes the i-th word.

**Definition 2** (charge keywords):

Under the guidance of legal experts, we designed a charge keyword list, as shown in the Table 2. Therefore, we can obtain the keywords for charges as $\text{K}={\{k}_{\text{1}},k_{\text{2}},...,k_{\left|K\right|}\}$, where $k_{i}$ denotes the i-th keyword and |K| is the total number of keywords. The maximum number of keywords corresponding to each charge is $k_{max}$.

**Table 2:** Some keywords for charges are displayed.

Charge	Keywords
Picking quarrels and provoking troubles	Randomly, batter, chase, abuse, damage, property, commotion, intimidate, Riot
illegally cultivating original plants of narcotics	Poppy, marijuana, cultivation, illegal, knowing, intentionally, drug, plant
Introducing graft	Introduction, bribery, state, functionaries, transmission, Graft, Facilitating
Abusing of power	Authority, abuse, overstepping, exceeding, Randomly, favoritism, fraud
Causing traffic casualties	Accident, driving, property, loss, speeding, overloading, red-light, no, license, reversing
Dangerous driving	Drunk, drinking, chase, speeding, cutting in, red light, overloading, driving, reversing
Kidnapping women and children	trafficking, women, children, kidnapping, trafficking, profit, coercion, deception, gain

**Definition 3**(Legal judgment prediction):

Legal judgment prediction (LJP) tasks like text classification, the purpose is to through the analysis of the legal text to predict the results of input [5]. In this paper, we keep cases with only one charge or only one legal article and perform single-label multi-class classification prediction.

we aim to train a model F(·) to predict judgment results of law articles$\;{\hat{y}}_{\text{1}}$, charges ${\hat{y}}_{\text{2}}$ and term of penalty ${\hat{y}}_{\text{3}}$ by making use of S(fact description) and K(keywords of charge) in a case, therefore LJP can be formalized as $F(S,K)=({\hat{y}}_{\text{1}},{\hat{y}}_{\text{2}},{\hat{y}}_{\text{3}})$

**Definition 4**(Category of charges):

For ease of representation, we define the categories of the charge prediction task as $\text{Q}={\{q}_{\text{1}},q_{\text{2}},...,q_{\left|Q\right|}\}$, here $q_{i}$ represents the i-th charge category and |Q| represents the total number of charge categories.

**Fig 2:**
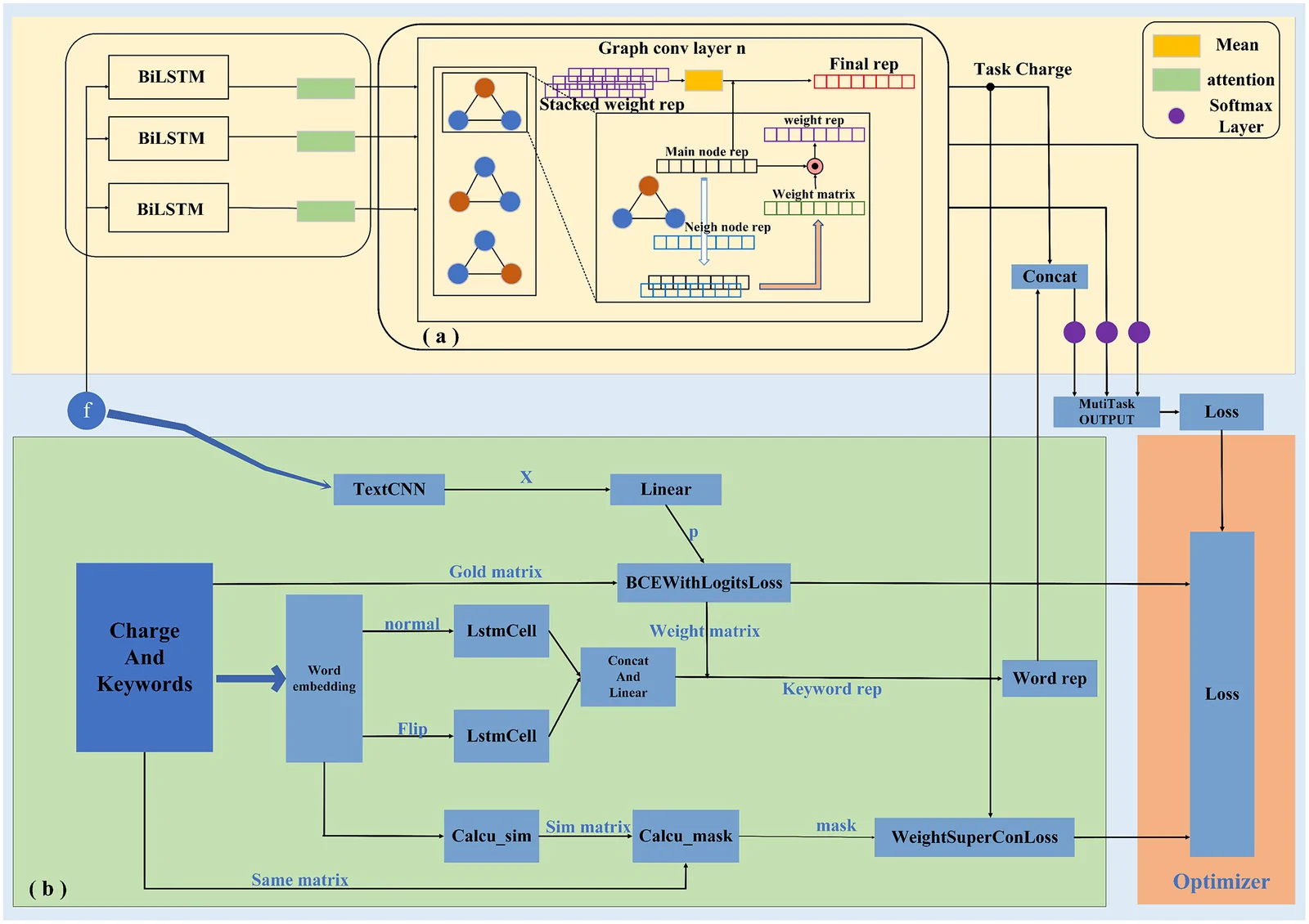
The specific architecture diagram of DTG-KCL. (a) is the dynamic task graph (DTG), (b) is the keyword-aware contrastive learning (KCL).

To model F(·), we have proposed a novel multi-task framework based on Dynamic Task Graph and Keyword-aware Contrastive Learning, named DTG-KCL, as shown in (Fig 2). Specifically, DTG-KCL comprises three key components: a basic encoding module—the attention-based fact encoding module—and two core modules: the dynamic task graph convolution module, and the keyword-aware contrastive learning module. we will provide a detailed description of the components in DTG-KCL.

2. Fact encoder with attention

According to the word sequence of a certain case $S\;=\;\{s_{\text{1}},s_{\text{2}},...,s_{n}\}$. For each word in S, we use the pre-trained word embeddings to convert each word $s_{i}$ into an embedding vector $e_{i}{\in R}^{d}$, where d is the dimension of the word embedding. Thus, we obtain a set of word embeddings corresponding to the factual description $\{e_{\text{1}},\;e_{\text{2}},...,\;e_{n}\}\in R^{n\times d}\;$.

Bi-LSTM [34] combines two independent LSTM [35] layers: one processes the input sequence from forward direction, and the other processes it from backward direction.

In this paper, we set up a Bi-LSTM encoder for each task t. Let ${\vec{h}}_{i}^{l}$ and ${\overset{\leftarrow }{h}}_{i}^{l}\;$denote the forward and backward hidden layer representations of the l-th layer corresponding to the word embedding $e_{i}$, respectively. They are computed as follows:


$$\begin{array}{c} \begin{array}{c} {\vec{h}}_{i}^{l}=\vec{lstm}\left(e_{i}\right),{\overset{\leftarrow }{h}}_{i}^{l}=\overset{\leftarrow }{lstm}\left(e_{i}\right) \end{array} \end{array}$$ (1)

We can obtain the final hidden layer vector $h_{i}^{l}\;=\;[\;{\vec{h}}_{i}^{l}\;;\;{\overset{\leftarrow }{h}}_{i}^{l}\;]\;(h_{i}^{l}\;\in \;R^{d_{l}})$, where the dimension of $d_{l}$ is the double of the LSTM hidden units. We input the word embeddings $\left\{e_{\text{1}},\;e_{\text{2}},...,\;e_{n}\right\}$ into the Bi-LSTM. The initial encoded representation of the certain case is the hidden states of the last layer (L) of the Bi-LSTM:


$$\begin{array}{c} \begin{array}{c} \left\{h_{\text{1}}^{L},h_{\text{2}}^{L},\ldots ,h_{n}^{L}\right\}=BiLSTM\left(e_{\text{1}},e_{\text{2}},\ldots ,e_{n}\right) \end{array} \end{array}$$ (2)

Considering that not all words in a sentence are equally important or contribute equally to the sentence vector, we apply an attention mechanism [36] to the initial encoding of the factual description. This allows us to learn the correlation weights between the hidden states at different time steps and the final state, and then aggregate the weights into the sentence embedding [37].


$$\begin{array}{c} \begin{array}{c} h_{stack}=\left\{h_{\text{1}}^{L},h_{\text{2}}^{L},\ldots ,h_{n}^{L}\right\}\in R^{n\times d} \end{array} \end{array}$$ (3)

$$\begin{array}{c} \begin{array}{c} u_{\alpha }=\text{tan}\text{h}\left({h_{stack}W}_{h}\right)h_{n} \end{array} \end{array}$$ (4)

$$\begin{array}{c} \begin{array}{c} C_{t}={h_{stack}}^{T}softmax\left(u_{\alpha }\right) \end{array} \end{array}$$ (5)

$W_{h}\in R^{d_{l}\times d_{l}}$ is trainable weight matrix, $h_{n}\in R^{d_{l}\times \text{1}}$ is obtained by Changing $h_{n}^{L}\in R^{d_{l}},u_{\alpha }\in \;R^{n\times \text{1}}$, $C_{t}{\in R}^{d_{l}\times \text{1}}$.

Thus, we obtain the initial encoded representation $C_{t}$ for task t. After performing this encoding operation three times, we obtain the initial encoded representations for the three subtasks: $C=\{C_{\text{1}},C_{\text{2}},C_{\text{3}}\}$.

3. Dynamic task graph convolution

To extract effective task relationships from $C_{t}$ of each task t, a straightforward approach is to preset the relationships between tasks in advance. However, in practice, judges may adopt different decision-making orders for different cases, and this simple method clearly cannot dynamically adjust task relationships based on the representation of each input case. Inspired by the popular Graph Convolution Operator (GCO) [5,38,39], we propose a Dynamic Task Relationship Extraction Graph Convolution Operator to efficiently and dynamically extract relationships between tasks.

In the graph convolution on the first layer, $v_{i}^{\text{1}}=C_{i},i=\text{1},\text{2},\text{3}$. In the l-th layer of graph convolution, the main node representation $v_{i}^{l}\in R^{d_{l}}$ and the neighbor node representation $v_{j}^{l}\in R^{d_{l}}$ are fused to generate the task edge fusion representation $v_{\left(i,j\right)}^{l}\in R^{d_{l}}$.


$$\begin{array}{c} \begin{array}{c} v_{\left(i,j\right)}^{l}=\left[v_{i}^{l},v_{j}^{l}\right]W_{a}+b_{a} \end{array} \end{array}$$ (6)

$$\begin{array}{c} \begin{array}{c} W_{\left(i,j\right)}^{l}=\left[Relu\left(Linear\left(\left[v_{i}^{l},v_{j}^{l}\right]\right)\right)\right]W_{m}+b_{m} \end{array} \end{array}$$ (7)

$$\begin{array}{c} \begin{array}{c} D_{\left(i,j\right)}^{l}=sigmoid\left(W_{\left(i,j\right)}^{l}\right) \end{array} \end{array}$$ (8)

$W_{\left(i,j\right)}^{l}$ is then processed to generate the **weight of dynamic task edge** $D_{\left(i,j\right)}^{l}\in (\text{0},\text{1})$. Subsequently, the weighted fusion representation is mean-pooled to obtain $v_{{agg}_{i}}^{l}\in R^{d_{l}}$.


$$\begin{array}{c} v_{{agg}_{i}}^{l}=\sum_{{j\in N}_{i}}\frac{D_{\left(i,j\right)}^{l}\cdot v_{\left(i,j\right)}^{l}}{\left|N_{i}\right|} \end{array}$$ (9)

$W^{a}\in R^{d_{l}}$and $b^{a}\in R^{d_{l}}$ are the trainable weight matrix and bias, respectively, $j\in N_{i}$ and $N_{i}$ is the set of neighbors of the representation of the i-th task (in this paper, all tasks initially form a fully connected graph, so $N_{i}$ is the set of task representations excluding $v_{i}^{l}$ itself).$W_{m}\in R^{d_{l}\times \text{1}}\;$and$\;b_{m}\in R^{d_{l}\times \text{1}}$ are the trainable weight matrix and bias, respectively.

Finally, through the residual connection [40], the representation of the main node $v_{i}^{l+\text{1}}\in R^{d_{l}}$ in the (l+1)-th layer of the graph convolution is obtained.


$$\begin{array}{c} \begin{array}{c} v_{i}^{l+\text{1}}=tanh\left(Linear\left(\left[v_{i}^{l}+v_{{agg}_{i}}^{l}\right]\right)\right) \end{array} \end{array}$$ (10)

Similar to GCO, our task relationship graph convolution operator also supports multi-layer stacking.

4. Keyword-aware contrastive learning
  (1) **The charge keywords representation construction**

Charge keyword library $\text{K}={\{k}_{\text{1}},k_{\text{2}},...,k_{\left|K\right|}\}$. For each word in K, we use the trained word vector model to convert each keyword $k_{i}$ into an embedding vector $e_{i}{\in R}^{d}$,where d is the dimension of the word embedding. Thus, we obtain a set of word embeddings corresponding to the keyword library $\{e_{\text{1}},\;e_{\text{2}},...,\;e_{\left|K\right|}\}\in R^{|K|\times d}$.

In this paper, we set up a Bi-LSTMCell encoder for the charge keywords:


$$\begin{array}{c} \begin{array}{c} {\vec{h}}_{i}=\vec{lstmCell}\left(e_{i}\right),{\overset{\leftarrow }{h}}_{i}=\overset{\leftarrow }{lstmCell}\left(e_{i}\right) \end{array} \end{array}$$ (11)

We can obtain the final hidden layer vector $h_{i}\;=\;[\;{\vec{h}}_{i}\;;\;{\overset{\leftarrow }{h}}_{i}\;]\;(h_{i}\;\in \;R^{d_{l}})$.


$$\begin{array}{c} \begin{array}{c} \left\{h_{\text{1}},h_{\text{2}},\ldots ,h_{\left|K\right|}\right\}=BiLSTMCell\left(e_{\text{1}},e_{\text{2}},\ldots ,e_{\left|K\right|}\right) \end{array} \end{array}$$ (12)

We reduce the dimension of $\left\{h_{\text{1}},h_{\text{2}},\ldots ,h_{\left|K\right|}\right\}\in R^{|K|\times d_{l}}$ through a linear layer to obtain the keyword library representation $\left\{{Rep}_{\text{1}},{Rep}_{\text{2}},\ldots ,{Rep}_{\left|K\right|}\right\}\in R^{|K|\times d}$.

(2) **Soft keywords representation**

Perform the TextCNN [1] operation on the set of word embeddings corresponding to the facts $\{e_{\text{1}},\;e_{\text{2}},...,\;e_{n}\}\in R^{n\times d}$ obtained in **Fact encoder with attention** to extract the key information $X\in R^{d}$ of the fact description.


$$\begin{array}{c} \begin{array}{c} X=TextCNN\left(e_{\text{1}},e_{\text{2}},\ldots ,e_{n}\right) \end{array} \end{array}$$ (13)

To capture the information from the key factual description information X that can guide keyword encoding, we first define the following linear function to perform an affine transformation on X, projecting it into the probability space. This is used to predict the most relevant keywords in the keyword library for the given case.


$$\begin{array}{c} \begin{array}{c} p=W_{g}X+b_{g} \end{array} \end{array}$$ (14)

$W_{g}\in R^{|K|\times d}$ and $b_{g}\in R^{\left|K\right|}$ represent the trainable weight matrix and bias respectively.

Then, we apply the Sigmoid operation to $p\in R^{\left|K\right|}$ to normalize the values into the range (0, 1), yielding a soft keyword weight matrix $W_{key}\in R^{\left|K\right|}$. Finally, we obtain the keyword representation for case guidance ${Rep}_{k}\in R^{d}$.


$$\begin{array}{c} \begin{array}{c} W_{key}=Sigmoid(p) \end{array} \end{array}$$ (15)

$$\begin{array}{c} \begin{array}{c} {Rep}_{k}=W_{key}\left\{{Rep}_{\text{1}},{Rep}_{\text{2}},\ldots ,{Rep}_{\left|K\right|}\right\} \end{array} \end{array}$$ (16)

For the loss of the auxiliary task of predicting keywords, we use the multi-class cross-entropy loss $L_{keyword}$.


$$\begin{array}{c} \begin{array}{c} L_{keyword}=-\left[y\cdot \text{log}(p)+(\text{1}-y)\cdot \text{log}(\text{1}-p)\right] \end{array} \end{array}$$ (17)

$y\in R^{\left|K\right|}$ refers to the matrix of the true labels of the charge keywords (where 0 represents the negative class and 1 represents the positive class). A case can correspond to multiple keywords of charge.

(3) **Calculate the WeightSupConLoss**

Based on the soft selection of keywords in the auxiliary task, this paper further employs supervised contrastive learning. The traditional SupConLoss [22] can only handle two classes. Therefore, this paper improves SupConLoss and proposes WeightSupConLoss (WSCL), which can handle three classes by applying weighted processing to hard negative samples.

In this paper, the cases within the same input batch are denoted as $B=\{b_{\text{1}},b_{\text{2}},\ldots ,b_{\left|B\right|}\}$, where $b_{i}$ represents the i-th case in the current batch and |B| denotes the batch size. The similarity of keywords is used to automatically identify which cases are “difficult negative samples.” Cases with the same charge are treated as positive sample pairs, cases with dissimilar keywords and different charges are treated as simple negative samples, and cases with similar keywords but different charges are treated as difficult negative samples. By weighting the difficult negative samples, the impact of difficult negative samples on the loss is increased, and WSCL is used to enlarge the distance between easily confused cases in the feature space.

${Same}_{batch}\in R^{\left|B|\times |B\right|}$ is the discrimination matrix of charge label for this batch, where 1 indicates the same labels and 0 indicates different labels.

Based on the set of keyword embeddings $\{e_{\text{1}},\;e_{\text{2}},...,\;e_{\left|K\right|}\}\in R^{|K|\times d}$ obtained from the **Soft keywords representation**, and the mapping between charges and their corresponding keywords, for charges that have fewer than $k_{max}$ keywords, this paper uses the mean vector for padding. Thus, we obtain $T\in R^{|Q|\times k_{max}\times d}$.

After performing mean pooling on T and calculating the cosine similarity, we obtain the charge similarity matrix ${Sim}_{keywords}\in R^{\left|Q|\times |Q\right|}$. Then, positions in ${Sim}_{keywords}$ that are greater than a threshold are set to 1, and those less than the threshold are set to 0, resulting in $Sim_{Binary}\in R^{|Q|\times |Q|}$ (a discrimination matrix of charge similarity determined by keywords similarity of charge). Then, we can obtain the similarity relationships between cases within the current batch, denoted as ${Sim}_{batch}\in R^{\left|B|\times |B\right|}$.

By combining ${Sim}_{batch}$ with ${Same}_{batch}$, we construct a ternary discrimination matrix $mask\in R^{\left|B|\times |B\right|}$, where cases with the same charge are labeled as 1, cases with dissimilar keywords and different charges are labeled as 0, and cases with similar keywords but different charges are labeled as −1.


$$\begin{array}{c} \begin{array}{c} L_{contrastive}=\text{WSCL}\left(\left[v_{B-charge}⨁v_{B-charge}\right],mask\right) \end{array} \end{array}$$ (18)

$v_{B-charge}=\{v_{c\text{1}},v_{c\text{2}},...,v_{c|B|}\}\in R^{|B|\times \text{1}\times d_{l}}$, $v_{ci}$ is the convolutional representation of the charge task that has undergone multiple layers of dynamic graph convolution. After a large number of experiments and verifications, we found that the method of constructing positive samples solely by rearranging words did not yield satisfactory results, and regard the sample itself as positive sample of the original sample. ⨁ is an operation of concatenation in the second dimension.

The formula of WSCL is specifically described as:


$$\begin{array}{c} \begin{array}{c} L_{supout}= \end{array} \end{array}$$

$$\begin{array}{c} \begin{array}{c} \sum_{i\in I}\frac{-\text{1}}{\left|P(i)\right|}\sum_{p\in P(i)}log\frac{\text{ex}\text{p}\left(v_{i}\cdot v_{p}/\tau \right)}{\sum_{\alpha \in A(i)}\text{ex}\text{p}\left(v_{i}\cdot v_{a}/\tau \right)+\sum_{\beta \in B(i)}W_{\beta }\text{ex}\text{p}\left(v_{i}\cdot v_{\beta }/\tau \right)} \end{array} \end{array}$$ (19)

In the above formula, i∈I≡{1…2N},N=|B|, represents the index of the sample after data augmentation. $v_{i}$ is the feature matrix of the current sample i. B(i) represents the set of indices of all difficult negative samples (i.e., for the difficult negative samples of i). A(i) represents the index set after excluding i and B(i). $P(i)\equiv \{p\in A(i)\;:{\tilde{y}}_{p}={\tilde{y}}_{i}\}$ represents the set of indices of all positive samples (i.e., samples of the same class as i) in the current batch. P(i) does not include the index i. $W_{\beta }$ is the weight of the difficult label, after conducting a sensitivity analysis, it is set to 1.5.

5. Multi-task prediction and joint loss

For each sub-task t**,** With the representation $v_{t}$ obtained through the graph convolution operation, we apply an affine transformation followed by softmax and obtain the final prediction as:


$$\begin{array}{c} \begin{array}{c} {\hat{y}}_{t}=softmax\left(v_{t}W_{t}+b_{t}\right) \end{array} \end{array}$$ (20)

Here, $W_{t}$ and $b_{t}$ are parameters specific to task t.

In particular, we carry out special processing on $v_{charge}$:


$$\begin{array}{c} \begin{array}{c} {\hat{y}}_{change}=softmax\left(Linear\left(\left[{Rep}_{k},v_{charge}\right]W_{t}\right)+b_{t}\right) \end{array} \end{array}$$ (21)

Here, ${\text{W}}_{\text{t}}$ and $b_{t}$ represent the trainable weight matrix and bias respectively.


$$\begin{array}{c} L\left({\hat{y}}_{t},y_{t}\right)=-\sum_{k=\text{1}}^{\left|Y_{t}\right|}y_{t,k}\text{lo}\text{g}\left({\hat{y}}_{t,k}\right) \end{array}$$ (22)

$$\begin{array}{c} L_{cls}=\sum_{j=\text{1}}^{\left|T\right|}\lambda_{t}L\left({\hat{y}}_{t},y_{t}\right) \end{array}$$ (23)

Here, $\lambda_{t}$ represents the weight factor for each sub-task t.


$$\begin{array}{c} \begin{array}{c} L=L_{cls}+\alpha \cdot L_{keyword}+\beta \cdot L_{contrastive} \end{array} \end{array}$$ (24)

Among them, $L_{cls}$ represents the classification cross-entropy loss for the three sub-tasks, $L_{keyword}$ is the loss for the auxiliary task of keyword prediction, $L_{contrastive}$ is the supervised contrastive learning loss, and α and β are the balancing parameters.

### Experiments

1. Dataset

**Fig 3:**
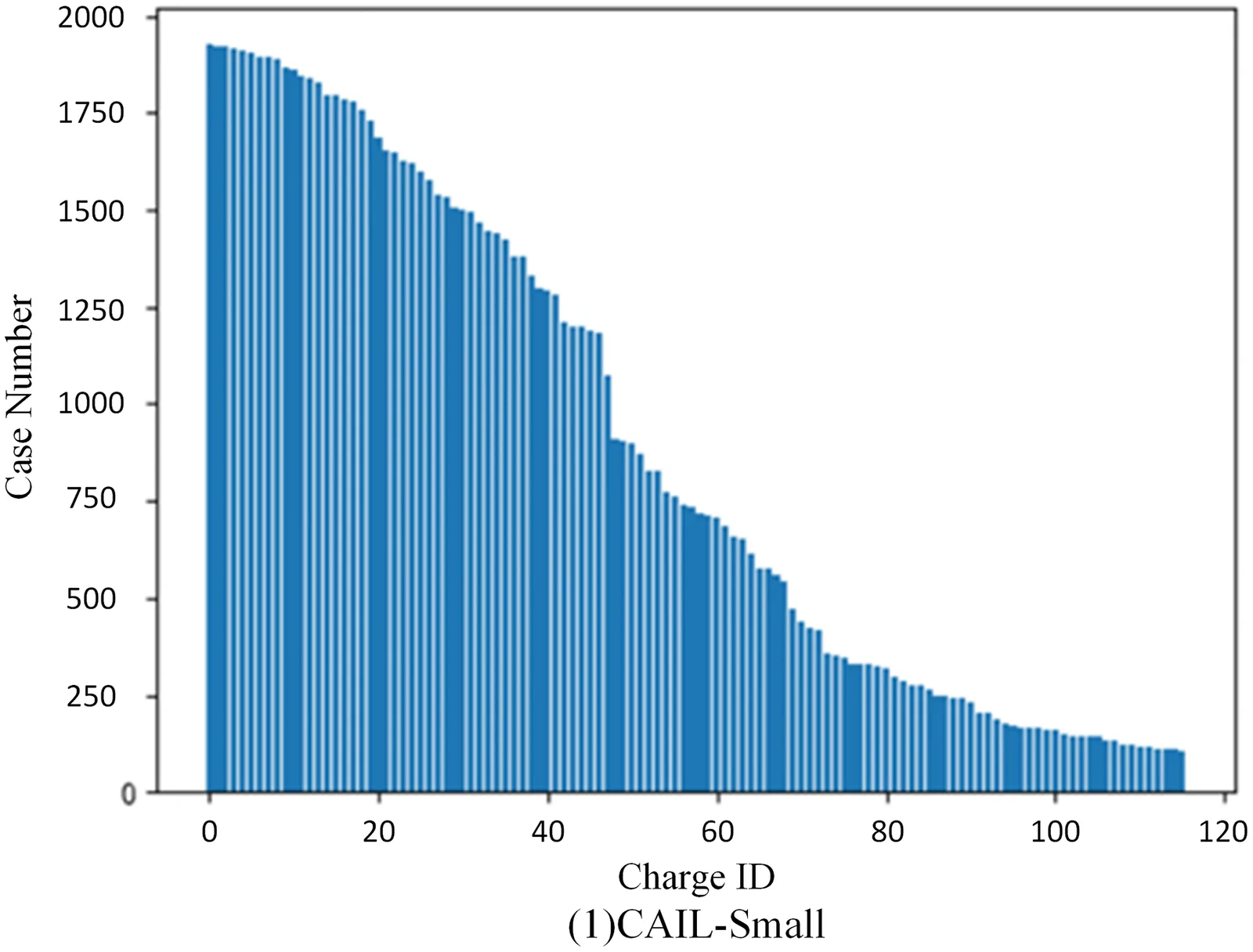
The x-axis is the charge ID, and the y-axis is the number of cases corresponding to that charge.

**Fig 4:**
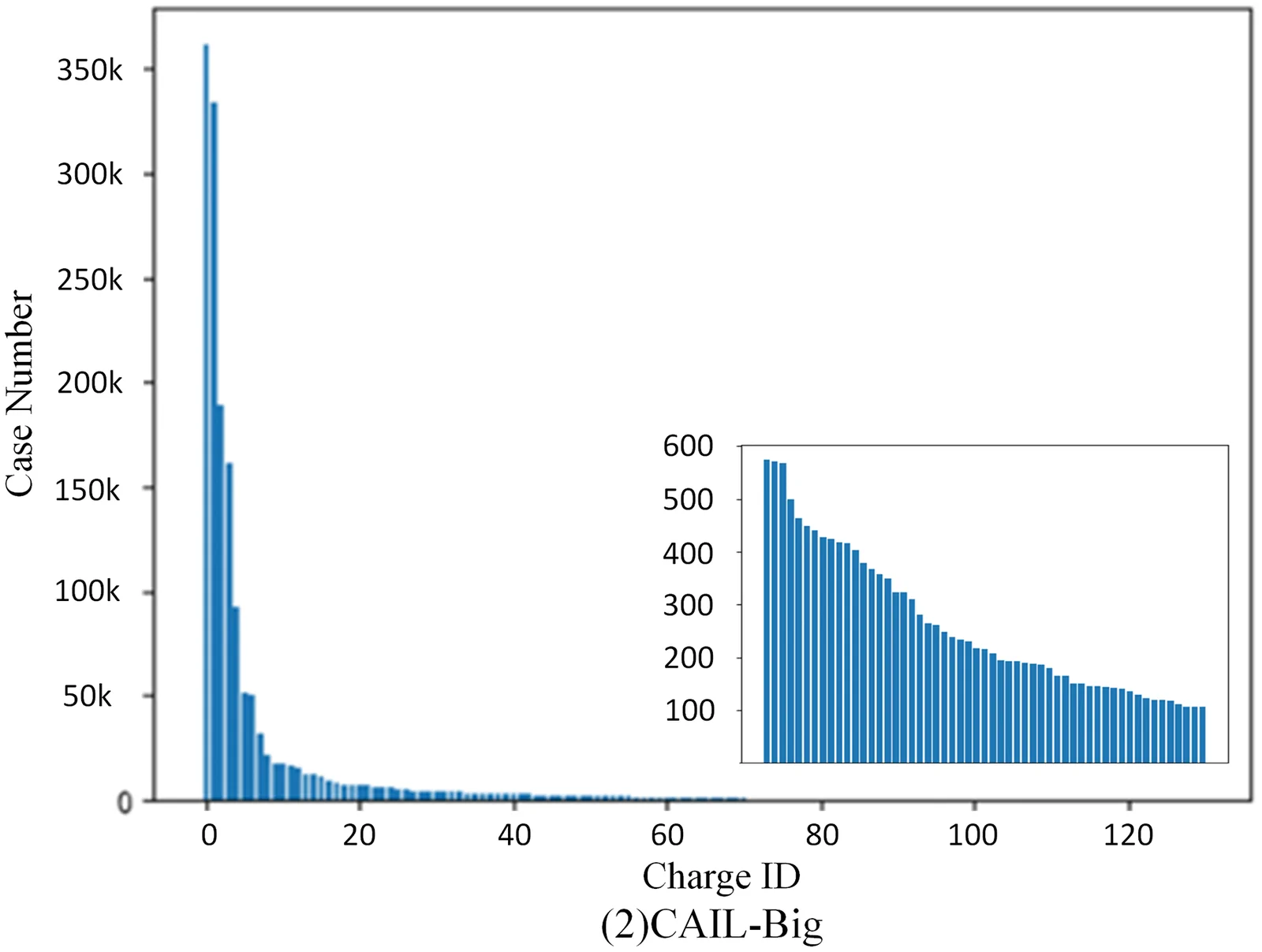
Since a small number of charges in the CAIL-Big dataset accounted for most cases, the number of cases corresponding to charges after ID 65 seemed to have almost vanished.

The CAIL2018 [41] dataset (The dataset can be obtained from https://github.com/china-ai-law-challenge/CAIL2018) consists of two sub-datasets, named CAIL-Small and CAIL-Big, respectively. We first conducted a statistical analysis on the CAIL-Small and CAIL-Big datasets. Since some cases are very uncommon, making it difficult to train models and make predictions for them, we filtered out uncommon charge and law article labels during data preprocessing, retaining only labels with a frequency greater than 100. As shown in Figs 3 and 4. we can clearly observe that the distribution of charge labels in the CAIL dataset is extremely imbalanced. We convert the regression prediction of penalty terms into a classification prediction. Therefore, the detailed statistics of the final experimental dataset are shown in the Table 3.

**Table 3:** Overview of the CAIL dataset.

Dataset	CAIL-Small	CAIL-Big
Training Set Cases	102,789	159,4368
Test Set Cases	25,088	185,856
Law Articles	97	118
Charges	116	130
Term of Penalty	11	11

2. Experimental setup

We used Thulac [33] to segment all Chinese words in the factual descriptions provided by CAIL. For the factual descriptions, we applied fixed word embedding model and the embedding dimension is 200. Meanwhile, we set the maximum vocabulary length for factual descriptions to 512 words. For factual descriptions with fewer than 512 words, we used mean vector padding; for those with more than 512 words, we used TF-IDF [6] to extract effective words. On CAIL-Small, the number of effective vocabulary words from the TF-IDF model is 20,000, while on CAIL-Big, the number of effective vocabulary words from the TF-IDF model is 50,000. In the word encoder module, the Bi-LSTM hidden unit size was set to 256. We also set the batch size for all models to 128, trained each model for 16 epochs, and evaluated on the test dataset. we set the weight factor λ for each subtask to 1, the weight factor λ for the keyword prediction auxiliary task to 1, and the weight factor λ for contrastive learning to 0.05. We adopted the Adam optimizer [42] with a learning rate of 0.001 to minimize the loss function of DTG-KCL.

3. Metrics

In this paper, we use accuracy (Acc), macro-precision (MP), macro-recall (MR), and macro-F1 (MF) as evaluation metrics. Here, following the work [3], we compute MP, MR, and MF1 by averaging the precision, recall, and F1 scores for each class.

4. Baseline methods

We will compare the proposed DTG-KCL model with the following methods:

TextCNN [1]: It is a model based on CNN for text classification.
BERT [43]: BERT is used to produce contextualized word embeddings. In this paper we adopted the Chinese BERT.
TopJudge [3]: is a topological multi-task learning model in LJP.
LADAN [5]: it uses a graph distillation operator to distinguish confusing verdicts with the learning of legal articles.
CL4LJP [27]: A supervised contrastive learning framework is used to handle LJP tasks.

5. Result and analysis

In this section, we conducted numerous experiments on CAIL-Small and CAIL-Big, and demonstrated the effectiveness of the proposed DTG-KCL model. We selected the three representative subtasks (i.e., the legal article prediction, charge and penalty term prediction) to compare.

(1) **Visual analysis**

Kernel Density Estimation (KDE) is a non-parametric method used for estimating the distribution of data. The abbreviation for the prediction task of law is “Law,” for the prediction task of charge it is “Charge,” and for the prediction task of term of penalty it is “Time”. (a, b) refers to the edge that points from node a to node b.

**Fig 5:**
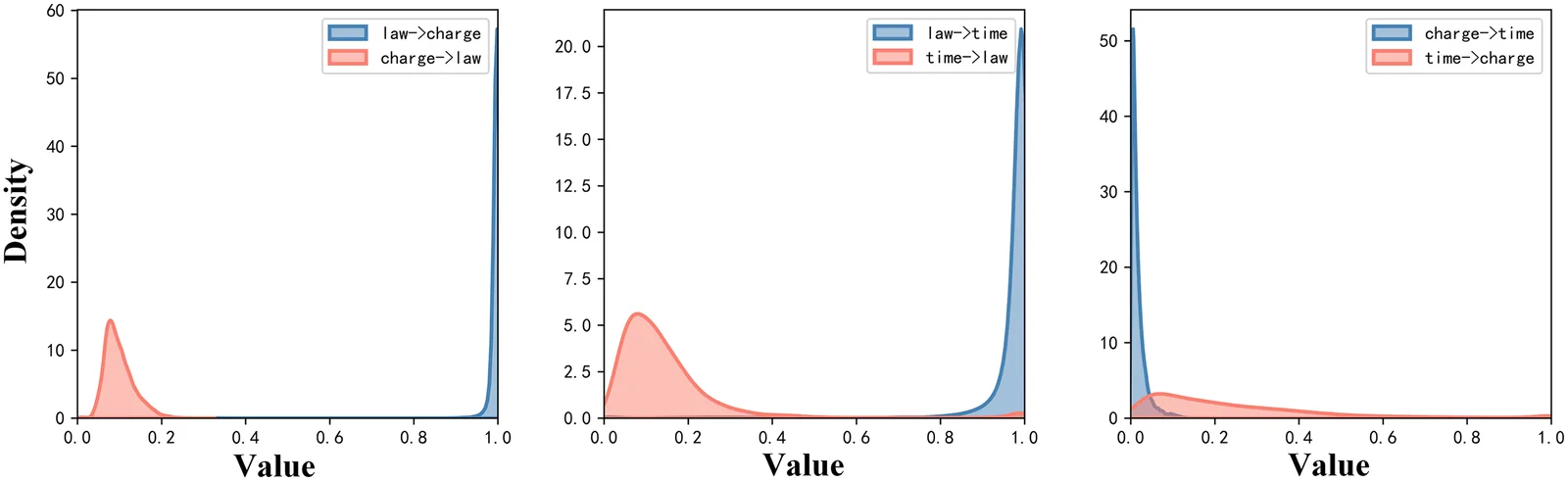
KDE graph of edge weights in the task graph.

We use KDE to analyze the edge weights in the dynamic task graph. As shown in (Fig 5), (1) the edge weights of (“Law”, “Charge”) and (“Law”, “Time”) are mostly distributed at 0.9, indicating that “Law” has a strong guiding effect on “Charge” and “Time.” Just as in a real trial, the judge first comes into contact with the facts of the case and the evidence (such as the methods of the crime, the consequences of the damage, the subjective intent, etc.), and the law articles determined based on this form the starting point for determining the charge and term of penalty.(2) The edge weights of (“Charge”, “Law”) and (“Charge”, “Time”) are mostly distributed between 0.05 and 0.1, suggesting that “Charge” has a very low guiding effect on “Law” and “Time.” In reality, judges rarely go back and reevaluate the facts based on a particular charge. (3) The edge weights of (“Time”, “Law”) are mostly distributed between 0.1 and 0.15, indicating that “Time” has a certain guiding effect on “Law”. The distribution of edge weights between (“Time”, “Charge”) is relatively balanced, indicating that the guiding role of “Time” on “Charge” is not constant but condition-dependent. In reality, after initially setting the sentence, judges sometimes review whether the factual determination matches the severity level of that sentence (for example, when sentencing to more than 10 years, they will re-examine whether any “particularly egregious” facts have been overlooked). This is a form of cognitive calibration, but its effect is limited.

**Fig 6:**
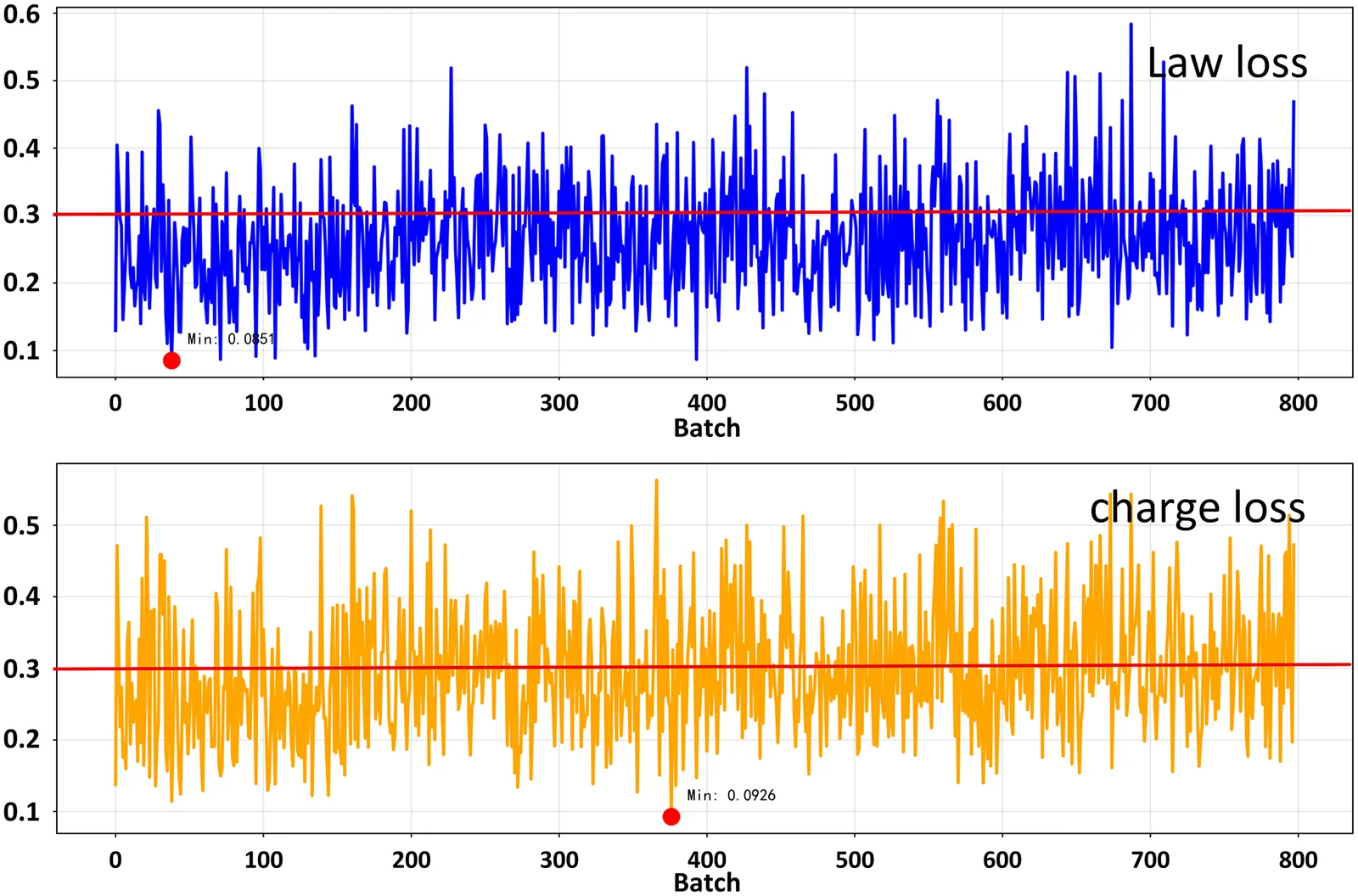
This is the training loss graph of the model without WSCL on the CAIL-small dataset at the 6th epoch of training.

**Fig 7:**
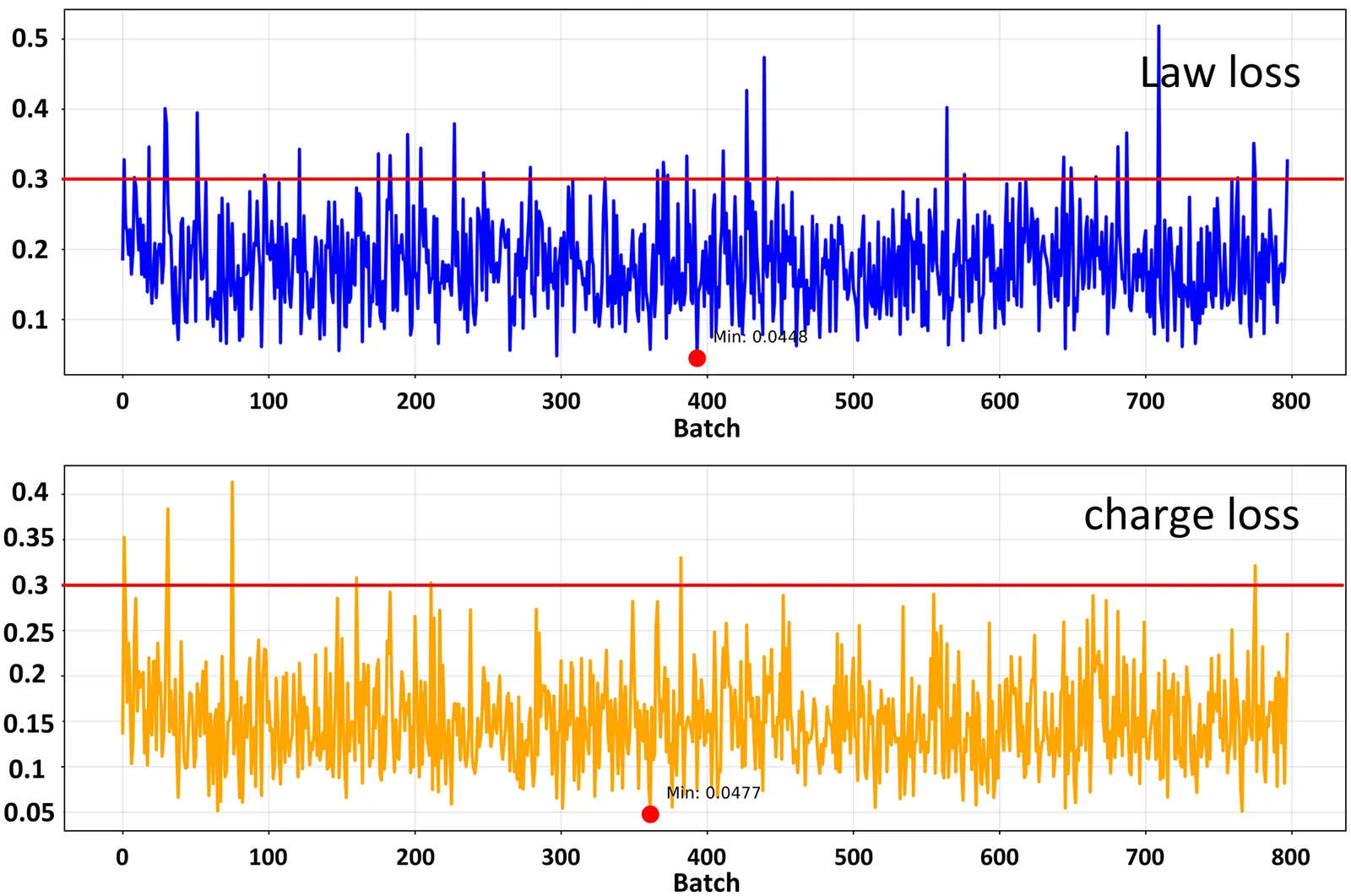
This is the training loss graph of the model with WSCL on the CAIL-small dataset at the 6th epoch of training.

After applying WeightSupConLoss (WSCL) based on the charge representation, as shown in the Figs 6 and 7, when the model has basically converged, under the same epoch, the loss of the law article prediction task and charge prediction task for the model with WSCL is lower than that of the model without WSCL, and the loss of charge prediction task remains stably lower than the loss of the law article prediction task. This fully demonstrates the effectiveness of our designed WSCL and the positive impact of WSCL on the law article prediction task.

**Fig 8:**
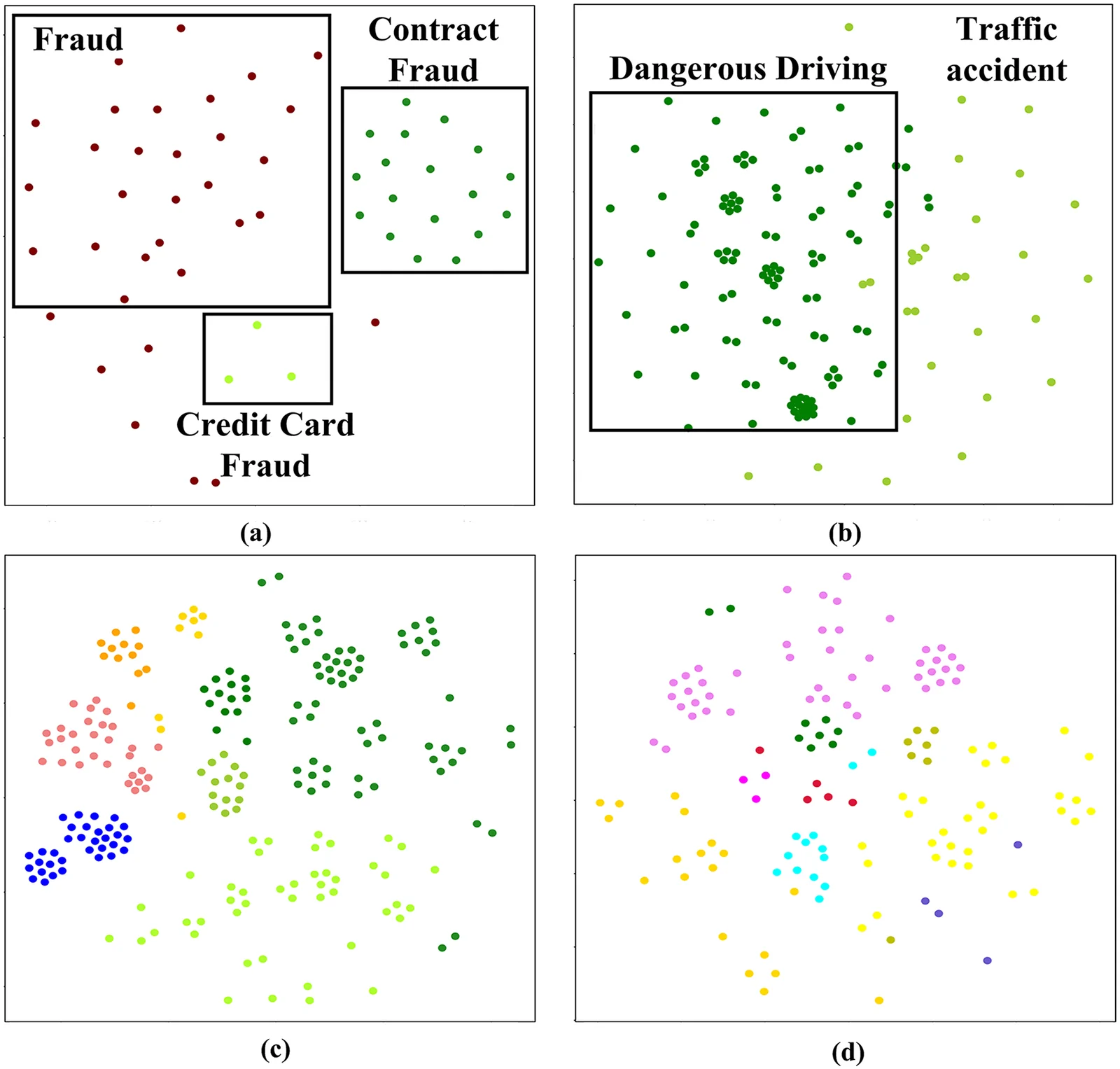
T-SNE visualization of charge representation.

We also used the t-SNE [44] dimensionality reduction technique to represent high-dimensional case representation in a two-dimensional space. The (Fig 8) shows the generated two-dimensional t-SNE plots. (a) demonstrates that the three charges—“fraud,” “contract fraud,” and “credit card fraud”—can be well distinguished by the model. (b) demonstrates that the pair of charges—“dangerous driving” and “traffic accident”—can also be well distinguished by the model. (c) and (d) show that when there are many easily confused charges, their distances in the feature space are larger, enabling the model to better distinguish them.

**Fig 9:**
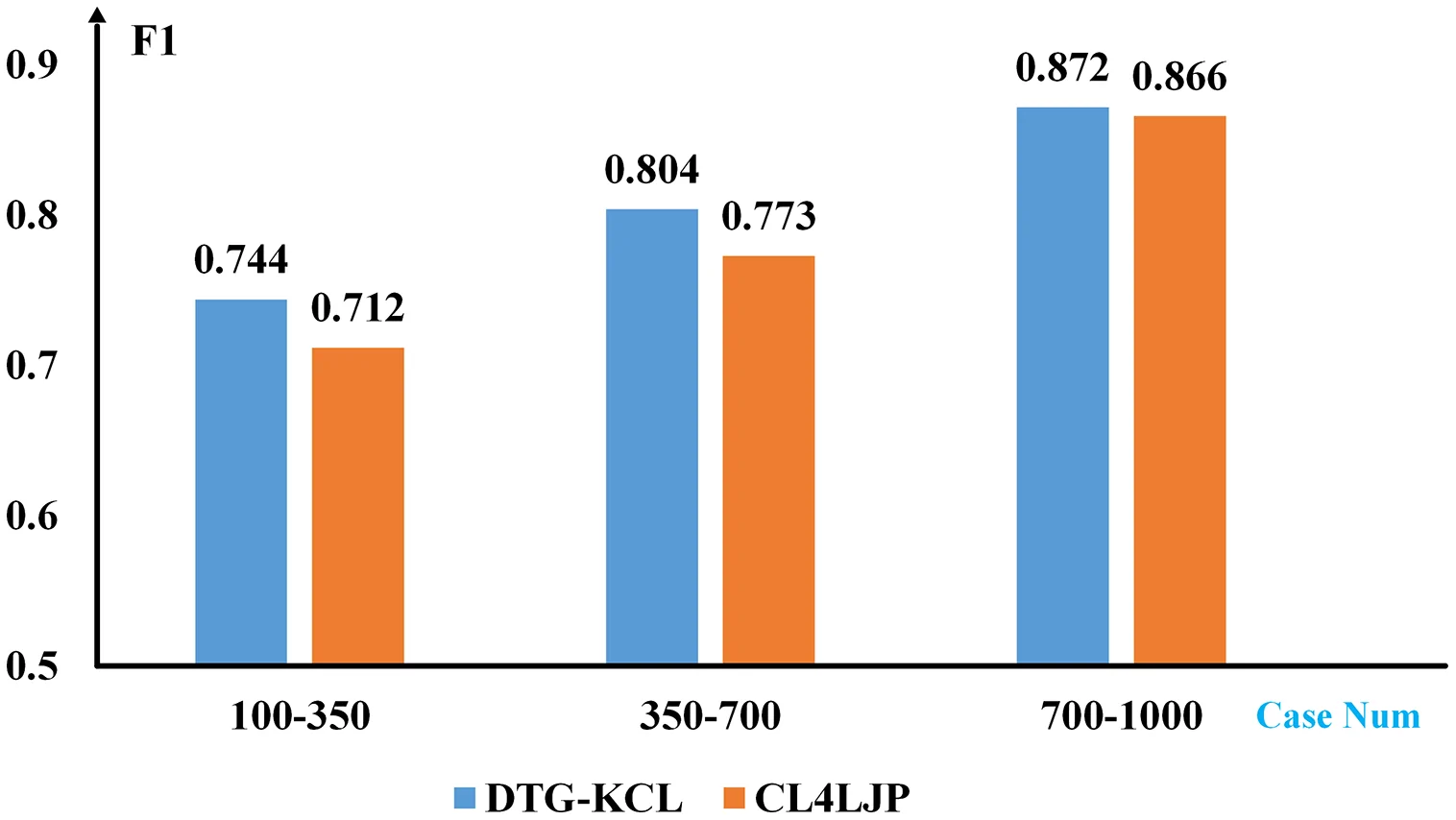
Comparison of the tail charge in F1.

We selected the charges from the CAIL-Big that had only a few samples (100–1000), and compared the classification results of DTG-KCL and CL4LJP on the test set. As shown in (Fig 9), we found that the F1 advantage of DTG-KCL in the charge prediction was more significant when the number of case samples was smaller.

(2) **Main experimental performance**

For the three sub-tasks in LJP, namely charge prediction (**Charges**), legal article prediction (**Law**), and term of penalty prediction (**Time**), **Table 4** summarizes the overall performance of our DTG-KCL compared to other baseline models on CAIL-Small, and **Table 5** summarizes the overall performance on CAIL-Big. Overall, DTG-KCL achieves significant improvements on both datasets, as reflected by all evaluation metrics.

**Table 4:** Experiment results on CAIL-Small dataset, where the best result for each metric is highlighted in bold.

Task	Law				Charges				Time			
model	Acc	MP	MR	F1	Acc	MP	MR	F1	Acc	MP	MR	F1
TextCNN	84.1	81.5	78.7	78.8	82.8	83.4	79.1	79.2	33.8	32.3	31.1	29.0
TopJudge	83.0	82.7	76.8	77.8	83.7	83.7	79.1	80.0	37.0	30.6	29.8	28.1
BERT	84.9	83.1	82.0	81.3	84.8	85.5	82.2	82.4	37.3	35.1	32.4	31.5
LADAN	86.0	85.7	**84.1**	**84.1**	86.2	85.8	83.9	84.0	38.3	35.1	32.8	32.7
CL4LJP	85.8	**86.0**	82.2	83.0	84.8	85.5	82.2	82.4	37.7	34.4	33.0	31.6
DTG-KCL	**86.8**	85.5	83.2	83.9	**88.3**	**88.0**	**86.4**	**86.8**	**38.8**	**36.4**	**34.8**	**35.1**

**Table 5:** Experiment results on CAIL-Big dataset, where the best result for each metric is highlighted in bold.

Task	Law				Charges				Time			
model	Acc	MP	MR	F1	Acc	MP	MR	F1	Acc	MP	MR	F1
TextCNN	96.0	85.5	75.1	77.3	95.6	86.0	74.6	77.5	58.2	48.9	41.9	42.6
TopJudge	96.2	86.5	75.3	77.7	95.6	89.4	75.3	78.9	58.3	48.8	43.4	43.3
BERT	96.2	85.3	77.0	78.8	95.9	88.5	77.7	79.9	58.3	47.6	42.2	43.5
LADAN	95.3	87.2	**81.4**	**83.5**	96.3	89.5	79.8	82.7	57.4	48.0	42.5	44.1
CL4LJP	96.4	86.5	78.5	80.5	96.2	89.4	79.6	82.4	59.3	49.5	44.3	45.6
DTG-KCL	**96.6**	**87.5**	80.1	82.4	**96.4**	**89.7**	**81.9**	**84.4**	**59.4**	**50.0**	**44.8**	**46.0**

Since both CAIL-Small and CAIL-Big are imbalanced datasets, we primarily compare F1 scores, which more objectively reflect the effectiveness of DTG-KCL and other baseline methods. We conducted the paired t-test on the results of multiple experiments, and the test results indicated that there was a significant difference within a 90% confidence interval.

- On both CAIL-Small and CAIL-Big, our DTG-KCL achieves significant improvements across all metrics for the three sub-tasks compared to some baseline models (TextCNN, TopJudge, and BERT). This fully demonstrates that our dynamic task relationship graph convolution effectively models the relationships among subtasks in a preliminary manner, and that the charge keyword-aware approach also effectively enhances the model’s ability to distinguish confusing charges.
- On CAIL-Small, compared to the CL4LJP model, DTG-KCL achieves improvements across all metrics for each sub-task. Specifically, the F1 score for the law article prediction task is 0.9% (±0.4%) higher than that of CL4LJP, the charge prediction task is 4.4% higher, and the term of penalty prediction task is 3.5% higher. On CAIL-Big, the F1 score for the law article prediction task is 1.9% higher than that of CL4LJP, the charge prediction task is 2% higher, and the term of penalty prediction task is 0.4% (±0.2%) higher. This demonstrates that the method of distinguishing simple negative samples from hard negative samples using the similarity of charge-corresponding keywords can better differentiate confusing charge labels.
- Interestingly, on the CAIL-Small dataset, for the charge prediction task and the term of penalty prediction task, the F1 scores of DTG-KCL are 2.8% and 2.4% higher than those of LADAN, respectively. On the CAIL-Big dataset, for the charge prediction task and the term of penalty prediction task, the F1 scores of DTG-KCL are 1.7% (±0.5%) and 1.9% (±0.3%) higher than those of LADAN, respectively. However, for the law article prediction task, the F1 score of DTG-KCL is 0.2% (±0.5%) lower than that of LADAN on CAIL-Small and the F1 score of DTG-KCL is 1.1% (±0.6%) lower than that of LADAN on CAIL-Big. Our model is only slightly inferior to LADAN in law article prediction. Therefore, we believe that the method of incorporating charge keywords enhances the semantic information between the case fact description and the charge labels, and increases the distance between easily confusable charge case representations in the feature space, giving it a clear advantage in the charge prediction task. However, our model has certain limitations in its predictive ability on law prediction. In the **Limitation and future work**, we have added a detailed noise analysis to explain this phenomenon.

(3) **Ablation study**

We conducted four ablation experiments on CAIL-Small to evaluate the contributions of the DTG module and the KCL module, aiming to demonstrate their importance within the attention fact encoding framework. (1) **w/o DTG**: In this variant, the dynamic task graph convolution module is removed from DTG-KCL, and the representation from the attention fact encoding module is fused with the keyword-aware module to obtain the final case representation. (2) **w/o KCL**: In this variant, the keyword-aware contrastive learning module is removed from DTG-KCL. Without keyword-aware contrastive learning, the representation obtained from the dynamic task graph convolution module serves as the final representation. (3) **w/o both**: In this variant, both the dynamic task graph convolution module and the keyword-aware contrastive learning module are removed from DTG-KCL, and the representation from the attention fact encoding module serves as the final case representation. (4) **TG-KCL**: In this variant, the dynamic weights of edge are removed from the DTG, and the unweighted neighbor representations are directly aggregated.

The Table 6 present the results of the ablation experiments. After removing DTG, the F1 scores for the law article prediction task, charge prediction task, and term of penalty prediction task decreased by 2.5%, 2.7%, and 2.6%, respectively, indicating that the DTG module effectively models the relationships among subtasks. After removing KCL, the F1 scores for the law article prediction task, charge prediction task, and term of penalty prediction task decreased by 0.9%, 5.8%, and 2.4%, respectively, demonstrating that the KCL module enhances the semantic information between the case fact description and charge labels, increases the distance between easily confusable charge case representations in the feature space, and has a certain positive impact on the law article prediction and term of penalty prediction tasks. After removing both modules, the F1 scores for the law article prediction task, charge prediction task, and term of penalty prediction task decreased by 2.5%, 9.5%, and 2.3%, respectively, highlighting the role of DTG-KCL in modeling task relationships and enhancing semantics within the attention fact encoding framework. After removing the dynamic weights of edge, All the metrics of the model have decreased, the dynamic edge weights not only enhance the predictive ability of the model to a certain extent, but also significantly improve the interpretability of the model.

**Table 6:** Comparison of different variants on the CAIL-Small dataset.

Task	Law			Charge			Time		
model	MP	MR	F1	MP	MR	F1	MP	MR	F1
**DTG-KCL**	**85.5**	**83.2**	**83.9**	**88.0**	**86.4**	**86.8**	36.4	**34.8**	**35.1**
**w/o DTG**	84.5	81.3	81.4	87.2	82.9	84.1	**36.8**	31.7	32.5
**w/o KCL**	85.4	82.7	83.0	84.1	80.7	81.0	35.1	32.8	32.7
**w/o both**	85.0	80.5	81.4	80.2	76.5	77.3	35.7	32.5	32.8
**TG-KCL**	85.1	82.6	83.2	87.5	86.1	86.4	36.2	34.3	34.8

(4) **Complexity analysis**

Each sample contains l words, the dimension of the hidden layer is d, the size of the batch is b, and the number of sub-tasks is t.

The time complexity analysis of the proposed model is presented in the Table 7.

**Table 7:** Time complexity analysis.

Time complexity	Basic encoder	DTG	KCL
Single sample (without WSCL)	O ($t*l*d^{\text{2}}$)	O$(t^{\text{2}}*d^{\text{2}}$)	O ($l*d^{\text{2}}$)+O$\left(d^{\text{2}}\right)$
Batch sample	O ($b*t*l*d^{\text{2}}$)	O$(b*t^{\text{2}}*d^{\text{2}}$)	O ($b*l*d^{\text{2}}$)+O$(b^{\text{2}}*d$) + O$\left(b*d^{\text{2}}\right)$

Here we assume that b=d=l, and since t≪d, t can be regarded as a constant, for example, in this paper t is 3. When only considering the training of a single sample, our overall complexity is approximately $O\left(d^{\text{3}}\right)$. When considering the scenario where batch samples are trained simultaneously, our overall complexity is approximately $O\left(d^{\text{4}}\right)$.

### Limitation and future work

**(The limitations of keyword library)** The charge keyword library used in this study mainly relies on the manual construction by domain experts [45,46]. The core advantage of this strategy lies in ensuring the accuracy of key terms and the compliance with law semantics, thereby providing high-quality prior semantic anchors for the model. However, this model has significant limitations at the following levels: (1) Conflict between static coverage and dynamic evolution; (2) Adaptation costs across judicial jurisdictions;(3) Lack of comparison verification with automatic/hybrid methods.

In the future, we will explore how to link law articles with charge keywords, and explore the methods for generating keywords using large language models, enabling the keywords to be integrated with the representation of law articles or to perform contrastive learning on the representation of law articles.

**(The limitations of law prediction)** some law articles can relate to multiple charges, some charges can correspond to multiple law articles (for example, Article 114 and Article 115 both involve “arson”). Therefore, when conducting comparative learning, the distance of “arson” in the feature space will be reduced, which is reflected in the loss of the comparative learning, thereby having a certain negative impact on the prediction of law article (law Article 114 and law Article 115). This also explains the performance deficiency of CL4LJP.

For contrastive learning, through our experiments, we have discovered that simply rearranging the order of words can damage the Semantics. In the future, we are considering introducing large language models to generate positive samples that are semantically similar but have different forms, instead of using traditional word rearrangement. Taking into account the correspondence between the charges and the law documents, we are considering incorporating more constraints in the contrastive learning.

Contrastive learning optimizes the discriminative power in the feature space, while the final evaluation metrics focus more on the classification decision boundary. Our improvement is limited. Part of the reason for this is that the improvement in the separability of the feature space does not lead to an equivalent increase in magnitude, especially in the case of severe class imbalance. However, LADAN achieves more direct benefits in the law article prediction by directly using the soft graph representation to assist in prediction.

For graph neural networks, in the future, we will explore shifting from vector-based methods to logic-based approaches to advance knowledge integration techniques, and design a graph neural network driven by an expert large model, which will process each case feature using the large model and generate enhanced graph node representations.

**(The limitations of advancement)** The baselines selected for this study (such as TopJudge, LADAN, CL4LJP, etc.) are mainly the classic and representative methods in LJP or in the NLP domain. Their advantages lie in the mature framework and stable results, which facilitate the verification of the core effectiveness of the proposed model. However, we fully acknowledge that the current comparison system does not fully cover the stronger benchmark models and large language models that have emerged in recent years. This is indeed our weakness, but the method we propose is a plug-and-play module rather than an indivisible system. Therefore, choosing a classic model as the starting point does not undermine the verification of the core effectiveness and we have already planned to introduce more novel methods as future work.

## Conclusion

In this paper, we propose DTG-KCL (Dynamic Task Graph and Keyword-aware Contrastive Learning). Specifically, we design a dynamic task relationship graph convolution method to encode task relationships from the perspective of each input case description. Subsequently, we improved the performance of multi-task prediction and the ability to distinguish between confusable charges by developing a more profound and novel keyword fusion mechanism, namely keyword-aware contrastive learning method. By introducing the relational extraction graph convolution algorithm and keyword-based contrastive learning, the model can better capture subtask relationships as well as the nuances and similarities among easily confused cases. Therefore, our model effectively improves the performance of the sub-tasks. Experiments on the CAIL-Small and CAIL-Big datasets show that DTG-KCL achieves significant performance improvements compared to traditional text classification methods and advanced LJP models.

## References

[1] Kim Y, editor. Convolutional neural networks for sentence classification. Proceedings of the 2014 conference on empirical methods in natural language processing (EMNLP). 2014.

[2] Chen H, Cai D, Dai W, Dai Z, Ding Y, editors. Charge-based prison term prediction with deep gating network. Proceedings of the 2019 Conference on Empirical Methods in Natural Language Processing and the 9th International Joint Conference on Natural Language Processing (EMNLP-IJCNLP). 2019.

[3] Zhong H, Guo Z, Tu C, Xiao C, Liu Z, Sun M, editors. Legal judgment prediction via topological learning. Proceedings of the 2018 conference on empirical methods in natural language processing. 2018.

[4] Yang W, Jia W, Zhou X, Luo Y. Legal judgment prediction via multi-perspective bi-feedback network. arXiv preprint arXiv:190503969. 2019.

[5] Xu N, Wang P, Chen L, Pan L, Wang X, Zhao J, editors. Distinguish confusing law articles for legal judgment prediction. Proceedings of the 58th annual meeting of the association for computational linguistics. 2020.

[6] Joachims T. A probabilistic analysis of the Rocchio algorithm with TFIDF for text categorization. 1996.

[7] Yang S, Tong S, Zhu G, Cao J, Wang Y, Xue Z, et al. MVE-FLK: A multi-task legal judgment prediction via multi-view encoder fusing legal keywords. Knowl-Based Syst. 2022;239:107960. doi: 10.1016/j.knosys.2021.107960

[8] Segal JA. Predicting Supreme Court cases probabilistically: The search and seizure cases, 1962-1981. Am Polit Sci Rev. 1984;78(4):891–900.

[9] LAUDERDALE BE, CLARK TS. The Supreme Court’s Many Median Justices. Am Polit Sci Rev. 2012;106(4):847–66. doi: 10.1017/s0003055412000469

[10] Liu Y-H, Chen Y-L, Ho W-L. Predicting associated statutes for legal problems. Inform Process Manage. 2015;51(1):194–211. doi: 10.1016/j.ipm.2014.07.003

[11] Liu Y, Wu Y, Zhang Y, Sun C, Lu W, Wu F, et al., editors. Ml-ljp: Multi-law aware legal judgment prediction. Proceedings of the 46th international ACM SIGIR conference on research and development in information retrieval. 2023.

[12] Hu Z, Li X, Tu C, Liu Z, Sun M, editors. Few-shot charge prediction with discriminative legal attributes. Proceedings of the 27th international conference on computational linguistics. 2018.

[13] Xu Z, Li X, Li Y, Wang Z, Fanxu Y, Lai X, editors. Multi-task legal judgement prediction combining a subtask of the seriousness of charges. China National Conference on Chinese Computational Linguistics. Springer; 2020.

[14] Li S, Zhang H, Ye L, Su S, Guo X, Yu H, et al. Prison Term Prediction on Criminal Case Description with Deep Learning. Comput Mater Contin. 2020;62(3):1217–31. doi: 10.32604/cmc.2020.06787

[15] Guo Y, Li Y, Ge F, Yu H, Wang S, Miao Z, editors. Legal judgment prediction via fine-grained element graphs and external knowledge. 2024 International Joint Conference on Neural Networks (IJCNN). IEEE; 2024.

[16] Zhang Y, Yang Q. A Survey on Multi-Task Learning. IEEE Trans Knowl Data Eng. 2022;34(12):5586–609. doi: 10.1109/tkde.2021.3070203 PMC10619966 37915376

[17] Cao J, Wang Y, He J, Liang W, Tao H, Zhu G. Predicting Grain Losses and Waste Rate Along the Entire Chain: A Multitask Multigated Recurrent Unit Autoencoder Based Method. IEEE Trans Ind Inf. 2021;17(6):4390–400. doi: 10.1109/tii.2020.3030709

[18] Yao F, Sun X, Yu H, Yang Y, Zhang W, Fu K. Gated hierarchical multi-task learning network for judicial decision prediction. Neurocomputing. 2020;411:313–26. doi: 10.1016/j.neucom.2020.05.018

[19] Qin W, Cao Z, Yu W, Si Z, Chen S, Xu J, editors. Explicitly integrating judgment prediction with legal document retrieval: a law-guided generative approach. Proceedings of the 47th international ACM SIGIR conference on research and development in information retrieval. 2024.

[20] Chen T, Kornblith S, Norouzi M, Hinton G, editors. A simple framework for contrastive learning of visual representations. International conference on machine learning. PmLR; 2020.

[21] He K, Fan H, Wu Y, Xie S, Girshick R, editors. Momentum contrast for unsupervised visual representation learning. Proceedings of the IEEE/CVF conference on computer vision and pattern recognition. 2020.

[22] Khosla P, Teterwak P, Wang C, Sarna A, Tian Y, Isola P. Supervised contrastive learning. Adv Neural Inform Process Syst. 2020;33:18661–73.

[23] Oord Avd, Li Y, Vinyals O. Representation learning with contrastive predictive coding. arXiv preprint arXiv:180703748. 2018.

[24] Gao T, Yao X, Chen D, editors. Simcse: Simple contrastive learning of sentence embeddings. Proceedings of the 2021 conference on empirical methods in natural language processing. 2021.

[25] Wu Z, Wang S, Gu J, Khabsa M, Sun F, Ma H. Clear: Contrastive learning for sentence representation. arXiv preprint arXiv:201215466. 2020.

[26] Meng Y, Xiong C, Bajaj P, Bennett P, Han J, Song X. Coco-lm: Correcting and contrasting text sequences for language model pretraining. Adv Neural Inform Process Syst. 2021;34:23102–14.

[27] Zhang H, Dou Z, Zhu Y, Wen J-R. Contrastive Learning for Legal Judgment Prediction. ACM Trans Inf Syst. 2023;41(4):1–25. doi: 10.1145/3580489

[28] Ribeiro de Faria J, Xie H, Steffek F. Information extraction from employment tribunal judgments using a large language model. Artif Intell Law. 2025. doi: 10.1007/s10506-025-09443-z

[29] Cheng L, Zhang S, Zhang H, Liu Q, Di B, Niyato D, et al. Large-Small Model Collaboration in Mobile Edge Networks With Heterogeneous Computational Resources. IEEE J Sel Areas Commun. 2026;44:2733–49. doi: 10.1109/jsac.2025.3644295

[30] Fan Y, Lin W, Chen R, Jiang H, Chai W, Wang J, et al. Gam-agent: Game-theoretic and uncertainty-aware collaboration for complex visual reasoning. Adv Neural Inform Process Syst. 2026;38:143569–629.

[31] Shi J, Zhao J, Wu X, Xu R, Jiang Y-H, He L. Mitigating reasoning hallucination through Multi-agent Collaborative Filtering. Exp Syst Appl. 2025;263:125723. doi: 10.1016/j.eswa.2024.125723

[32] Jiang C, Yang X. AgentsBench: A Multi-Agent LLM Simulation Framework for Legal Judgment Prediction. Systems. 2025;13(8):641. doi: 10.3390/systems13080641

[33] Sun M, Chen X, Zhang K, Guo Z, Liu Z. Thulac: An efficient lexical analyzer for chinese. Retrieved Jan. 2016;10:2022.

[34] Schuster M, Paliwal KK. Bidirectional recurrent neural networks. IEEE Trans Signal Process. 1997;45(11):2673–81. doi: 10.1109/78.650093

[35] Hochreiter S, Schmidhuber J. Long short-term memory. Neural Comput. 1997;9(8):1735–80. doi: 10.1162/neco.1997.9.8.1735 9377276

[36] Bahdanau D, Cho K, Bengio Y. Neural machine translation by jointly learning to align and translate. arXiv preprint arXiv:14090473. 2014.

[37] He C, Tan T-P, Zhang X, Xue S. Knowledge-Enriched Multi-Cross Attention Network for Legal Judgment Prediction. IEEE Access. 2023;11:87571–82. doi: 10.1109/access.2023.3305259

[38] Kipf TN, Welling M. Semi-supervised classification with graph convolutional networks. arXiv preprint arXiv:160902907. 2016.

[39] Hamilton W, Ying Z, Leskovec J. Inductive representation learning on large graphs. Adv Neural Inform Process Syst. 2017;30.

[40] He K, Zhang X, Ren S, Sun J, editors. Deep residual learning for image recognition. Proceedings of the IEEE conference on computer vision and pattern recognition. 2016.

[41] Xiao C, Zhong H, Guo Z, Tu C, Liu Z, Sun M, et al. Cail2018: A large-scale legal dataset for judgment prediction. arXiv preprint arXiv:180702478. 2018.

[42] Kingma DP, Ba J. Adam: A method for stochastic optimization. arXiv preprint arXiv:14126980. 2014.

[43] Devlin J, Chang M-W, Lee K, Toutanova K, editors. Bert: Pre-training of deep bidirectional transformers for language understanding. Proceedings of the 2019 conference of the North American chapter of the association for computational linguistics: human language technologies, volume 1 (long and short papers). 2019.

[44] Van der Maaten L, Hinton G. Visualizing data using t-SNE. J Mach Learn Res. 2008;9(11).

[45] Liu Q, Yu H, Wang Q, Xu Q, Li J, Zou Z, et al. Legal knowledge infusion for large language models: A survey. Inf Fusio. 2026;125:103426. doi: 10.1016/j.inffus.2025.103426

[46] Zhang Y, Wei X, Yu H. HD-LJP: A Hierarchical Dependency-based Legal Judgment Prediction Framework for Multi-task Learning. Knowl-Based Syst. 2024;299:112033. doi: 10.1016/j.knosys.2024.112033

